# Clinical and biochemical assessment of the effect of topical use of coenzyme Q10 versus topical corticosteroid in management of symptomatic oral lichen planus: randomized controlled clinical trial

**DOI:** 10.1186/s12903-023-03206-5

**Published:** 2023-07-21

**Authors:** Mostafa Abdelsamie, Fat’heya Zahran, Amal A. Hussine, Olfat Shaker, Abdulaziz Mohsen Al-mahallawi

**Affiliations:** 1 Oral Medicine, Faculty of Dentistry, Cairo University, Cairo, Egypt; 2 Oral Medicine, Faculty of Dentistry, Cairo University, Cairo, Egypt; 3 Oral Medicine, Faculty of Dentistry, Cairo University, Cairo, Egypt; 4 Medical Biochemistry and Molecular Biology, Faculty of Medicine, Cairo University, Cairo, Egypt; 5 Department of Pharmaceutics and Industrial Pharmacy, Faculty of Pharmacy, Cairo University, Cairo, Egypt; 6 School of Life and Medical Sciences, University of Hertfordshire Hosted By Global Academic Foundation, New Administrative Capital, Cairo, Egypt

**Keywords:** Oral lichen planus, Oxidative stress, Corticosteroids, Antioxidants, CoQ10, Malondialdehyde, Saliva

## Abstract

**Background:**

Oral lichen planus (OLP) is a chronic mucocutaneous immunologically mediated condition that has a great adverse effect on oral functions. Corticosteroids are still the first drugs of choice used in the treatment of OLP; however, they have extensive medical side effects. The present study was carried out to assess the clinical therapeutic effect of the topical use of coenzyme Q10 (coQ10 or ubiquinol) versus topical corticosteroids in the management of symptomatic OLP and to determine whether the effect, if any, was due to the powerful antioxidant activity of coQ10.

**Subjects and methods:**

We performed a randomized, double blinded controlled trial at the Faculty of Dentistry, Cairo University, Egypt. The study was conducted on 34 patients suffering from symptomatic OLP. Patients were randomly divided into two groups: intervention group (I),who received topical CoQ10 in the form of mucoadhesive tablets (40% CoQ10) 3 times daily for one month and control group (II),who received topical corticosteroid (kenacort in Orabase: triamcinolone acetonide 0.1% 5-g adhesive paste – dermapharm), 4 times daily for one month. Patients were evaluated at one-week intervals using the clinical parameters (score) of pain (VAS) and lesion size. Additionally, salivary levels of malondialdehyde (MDA) were detected in both groups before and after treatment using ELISA. All recorded data were analysed using independent t test, ANOVA followed by Bonferroni post hoc test for lesion size and salivary level of MDA data and Mann–Whitney U test and Friedman test for VAS data.

**Results:**

Both groups showed a significant reduction in pain and the size of the lesions (*p* ≤ 0.05) with no statistically significant difference between them (*p* > 0.05), and this clinical improvement was associated with a reduction in the salivary levels of MDA in both groups.

**Conclusions:**

The topical use of CoQ10 mucoadhesive tablets was as effective as the topical use of triamcinolone acetonide, and its clinical effect was associated with a reduction in the salivary level of MDA.

**Trial registration:**

The study protocol was registered at www.clinicaltrial.gov (NCT04091698) and registration date: 17/9/2019.

## Introduction

Oral lichen planus (OLP) is a relatively common chronic mucocutaneous inflammatory immune-mediated disease of the oral mucosa [28], it affects middle-aged females twice as much as males [14], with an estimated general population prevalence of 0.89% [28], and has recently been categorized as an oral potentially malignant disorder by the World Health Organization (WHO) [60]. It has a variety of clinical forms, which may occur alone or in various combinations [20, 52], where the atrophic and erosive forms are the most severe and are introduced to oral medicine clinics with severe burning sensation affecting different oral functions [38].

On the other hand, oral lichenoid lesions (OLLs) are a term used to identify conditions that are clinically and histopathologically similar to OLP but with identifiable, either local or systemic causes such as numerous medications, various dental materials (mercury-containing amalgam restorations), and graft versus host disease (GVHD). Compared to the traditional signs of OLP, OLLs tend to be unilateral with histological examination showing more diffuse lymphocytic infiltration with more eosinophils, plasma cells, and colloid bodies. In addition, it resolves once the cause is removed [24].

Multiple factors [16] and immunological responses [9] are implicated in the pathogenesis of OLP. Among these, oxidative stress (OS) is implicated in both OLP pathogenesis and carcinogenic potential [29]. Higher salivary levels of reactive oxygen species (ROS), lipid peroxidation, nitric oxide, and nitrite support this theory [33], along with the obvious decrease in total antioxidant activity and an increased level of salivary oxidative markers in OLP patients compared to controls [53]. OS is defined as a disruption in the balance of pro-oxidant/antioxidant processes in biological organisms [25]. It is produced by an excess of ROS or a breakdown in antioxidant functions. ROS can harm human cells by causing protein, carbohydrate, lipid, and nucleotide damage [4].

In OLP lesions, ROS exacerbate inflammatory conditions linked to immunological pathways through the activation of NF-kB (nuclear factor kappa-light-chain-enhancer of activated B cells), a protein complex that regulates proinflammatory gene transcription, such as interleukin 2 (IL-2), tumor necrosis factor-alpha (TNF-α), MHC class 1 gene, and IL-2 receptor gene [2, 30]. TNF-α promotes T-lymphocyte recruitment by upregulating matrix metalloproteinase (MMP), which disrupts basement membrane integrity [46].

A variety of treatments are used in the management of OLP [16, 27]. Among these treatments, corticosteroids are the gold standard treatment for OLP due to their anti-inflammatory and immunomodulatory actions through different mechanisms, including decreased leukocyte exudates into inflamed areas through the inhibition of vasodilation and vascular permeability, repressed transcription of many genes encoding proinflammatory cytokines, including NF-κB, suppressed adhesion molecules expression, such as ICAM-1 and VCAM-1, and regulation of Th1 responses and autoimmunity through their direct effect on T cells with stimulation of IL10 secretion [12]. However, the long term use of steroids showed different side effects ranging from atrophy of the oral mucosa or secondary candidiasis, associated with the topical use of corticosteroid therapy [31], to more serious systemic side effects, such as hypertension, osteoporosis and adrenal insufficiency, associated with the systemic administration of corticosteroids [15, 62], resulting in a continuing search for safer and more effective therapies.

Co enzyme Q10 is a lipid-soluble endogenous antioxidant compound due to its ability to scavenge free radicals such as superoxide anion (O2•), hydrogen peroxide (H2O2) and hydroxyl radical (OH•) [7]. It also augments the function of other endogenous antioxidants, such as α-tocopherol (vitamin E) and ascorbate (vitamin C) [36, 57]. In addition, it enhances other antioxidant enzymes, such as superoxide dismutase (SOD), catalase (CAT) and glutathione peroxidase [10]. In addition to the antioxidant role of CoQ10, it has an anti-inflammatory role through its suppression to the gene expression of NFκB1 and the overproduction of proinflammatory cytokines such as TNF-α and interleukin-6 [8, 18],Furthermore, it promotes the expression of anti-inflammatory cytokines such as IL-10 [23], thus promoting tissue regeneration and wound healing [51, 61].

## Subjects and methods

### Study design

The present study is a randomized controlled clinical trial (two parallel groups) with an allocation ratio of 1:1. The number of patients was equal in each group. The study was conducted following the principles of the Helsinki Declaration and was approved by the Research Ethics Committee of the Faculty of Dentistry, Cairo University (code: 19923).The protocol was registered at www.clinicaltrial.gov (NCT04091698).

### Study participants

The patients were recruited from the Diagnostic Center, as well as the Clinics of the Oral Medicine and Periodontology Department, at the Faculty of Dentistry, Cairo University, during the period from September 2019 to February 2022. According to specific inclusion and exclusion criteria**.**

### Inclusion criteria

1. Patients were more than 18 years old.
2. Patients were free from any systemic disease according to the detailed questionnaire of the modified Cornell Medical Index [43].
3. Patients clinically diagnosed by a dermatologist and oral medicine specialist as suffering from OLP.
4. Patients who agreed to the biopsy in undiagnosed cases**.**
5. Clinical and histopathological criteria were used according to modified WHO diagnostic criteria for OLP [59].
6. Patients who were willing to participate in this study (who agreed to give informed consent) and had the ability to complete the study.

### Exclusion criteria

1. Patients taking systemic drugs such as systemic steroids, or other immunosuppressive therapies for at least 8 weeks prior to the study.
2. Patients treated with any oral topical medications for at least four weeks prior to the study.
3. Patients receiving any medication either topical or systemic that could cause lichenoid reaction during the 3 months before the study.
4. Patients with suspected restoration or drug-related lichenoid lesions.
5. Pregnant and lactating females.

### Study interventions

**Fig. 1:**
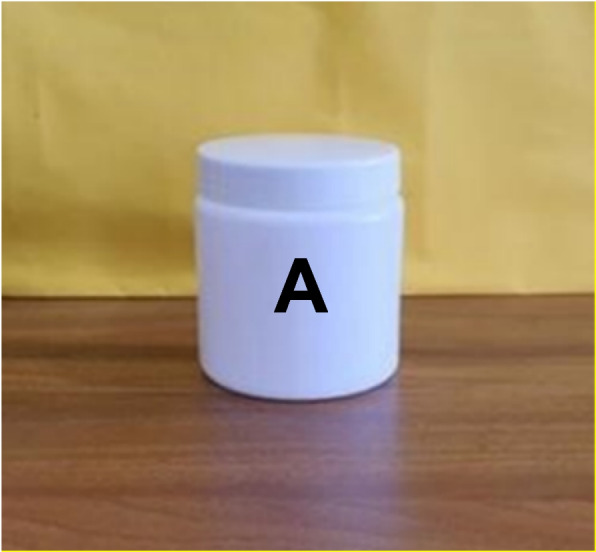
Opaque sealed jar containing mucoadhesive CoQ10 tablets

The present study was conducted on 34 patients suffering from symptomatic OLP. Patients were randomly divided into two groups and received both treatments in the form of opaque sealed jars (Jar A) for adhesive tablets (Fig. 1) and (Jar B) for triamcinolone paste (Fig. 3):

#### Group I (intervention group)

Seventeen participants received topical coenzyme Q10 (ubiquinol) in the form of mucoadhesive tablets, (Fig. 1), 3 times daily for one month. All patients were instructed to apply slight pressure for 1 min on the entire surface of the tablet using their finger and then let it dissolve without peeling it off. They were also instructed to avoid bringing their teeth into contact with the tablets, to avoid chewing or excessive jaw movements, and to avoid eating or drinking for at least 1 h following application of the tablet [11]. They were also instructed to apply the tablet on a single lesion that was the most painful lesion for the patient, and found to be related to the buccal mucosa in most cases.

#### CoQ10 mucoadhesive tablet preparation

**Fig. 2:**
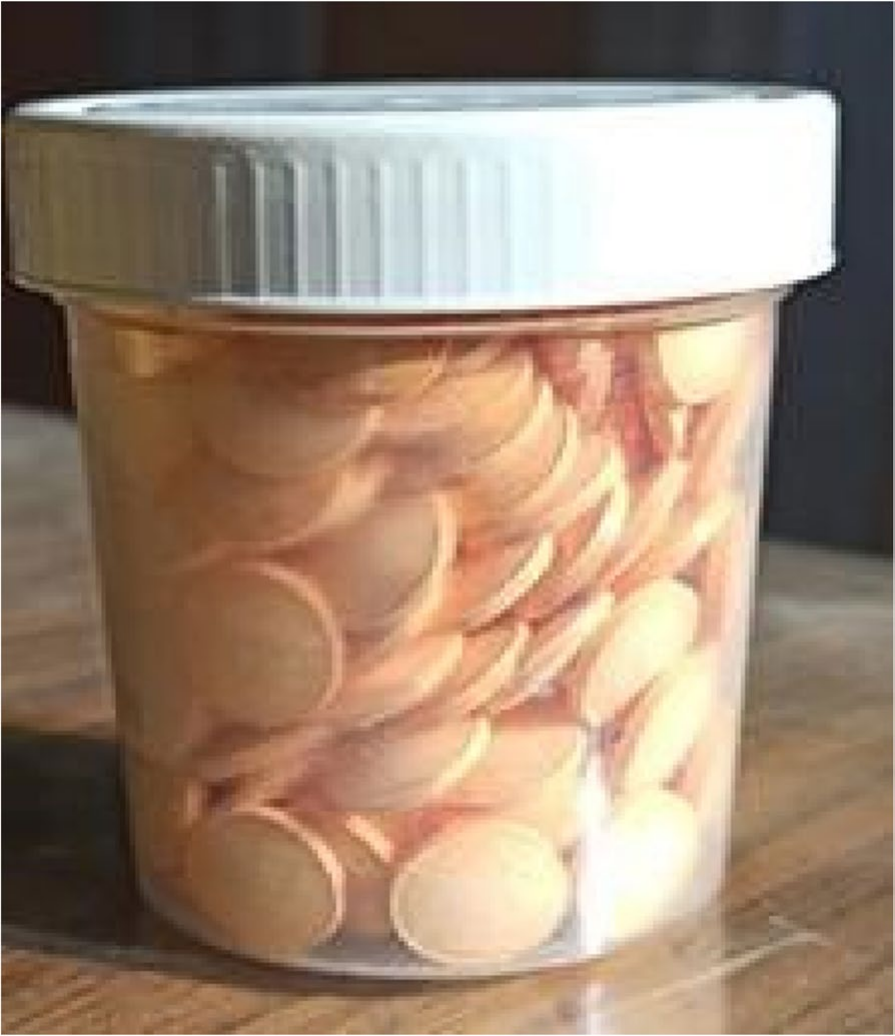
Mucoadhesive CoQ10 tablets

The tablets were prepared using 120 mg coQ10 powder (in reduced form which is the antioxidant form) [41], mixed with mucoadhesive polymer 120 mg carbapol [42], and 60 mg anhydrous lactose [21], using a bench scale powder mixer continuously for 10 min. The components of each tablet were fed manually into a 13 mm die and compressed using a constant compression force to produce tablets (40% coQ10 concentration) with a 13 mm surface area and hardness of 10 kgf (Fig. 2).

#### Group II (control group)

**Fig. 3:**
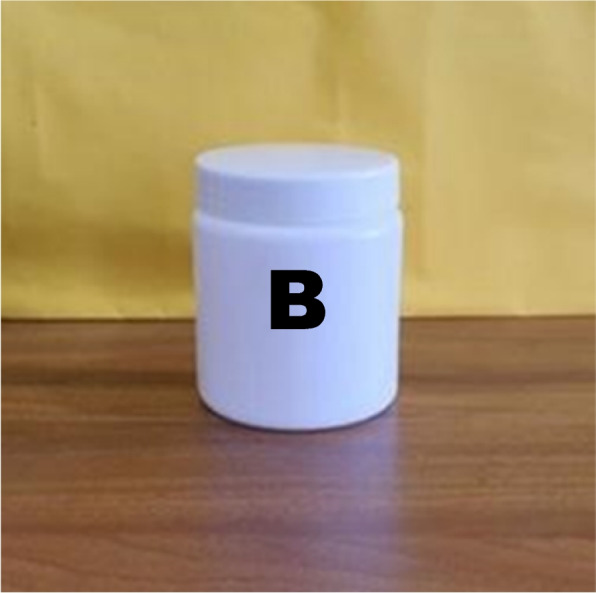
Opaque sealed jar containing triamcinolone acetonide paste

Seventeen participants received topical corticosteroid (kenacorte in Orabase: triamcinolone acetonide 0.1% 5-g adhesive paste – dermapharm), (Fig. 3), 4 times daily for one month [26]. All patients were instructed to apply a thin layer using a finger or cotton tip applicator, considering not to eat, drink, or speak for at least 1 h. Miconazole 2% topical antifungal (Miconaz® oral gel: Miconazole 2 g per 100 gm) (Medical Union Pharmaceuticals—MUP—Egypt) was applied after a 4 week follow- up period to avoid secondary candidiasis in this group [22].

### Study outcomes

1. Primary outcome
  1.1. Pain measurement using the Visual Analogue Scale: according to Maxwell [32]
  All patients were asked to define their level of pain and discomfort by using a numerical rating from 0 to 10 (11-points), with 0 indicating "no pain", 1 to 3 indicating mild pain, 4 to 6 indicating moderate pain, 7 to 9 indicating sever pain and 10 indicating "extremely painful".
  1.2. Clinical improvement of the lesion, according to Thongprasom et al. [56]
     0 = no lesion
     1 = white striae only
    2  = white striae and atrophic ≤ 1 cm^2^
    3  = white striae with atrophic > 1 cm^2^
     4 = white striae with erosion ≤ 1 cm^2^
     5 = white striae with erosion > 1 cm^2^
    The clinical score for each patient was calculated by recording a score for each lesion in the oral cavity separately using a graduated periodontal probe, and then calculating the average of these scores.
2. Secondary outcome
  2.1. Change in salivary level of malondialdehyde detected at baseline and after treatment (after 4 weeks) using ELISA.
  2.2. Change in Clinical global impression scale detected from baseline to the end of treatment after 4 weeks, in which the patients rated overall change in OLP symptoms during the treatment period (patient global impression of change; PGI-C), choosing 1 of 7 answers ranging from “very much better” to “very much worse.” 1 = very much better/improved, 2 = much better/improved, 3 = a little better/improved, 4 = no change, 5 = a little worse, 6 = much worse, and 7 = very much worse.

### Saliva sample collection

Whole unstimulated saliva (WUS) was collected between 8 am to 1 pm using standard techniques according to Navazesh [37]. At the time of saliva collection, lesions were actively symptomatic, and subjects were asked not to eat, brush their teeth, or use mouth rinse at least 2 h prior to salivary sample collection on that day. Samples were obtained by requesting subjects to swallow first, tilt their heads forward, and expectorate 10 mL of unstimulated whole saliva into a sterile centrifuge tube. After collection, the saliva was immediately centrifuged for 2 min at 10,000 × g and the clarified supernatant was filtered through a 0.45 μm low protein binding membrane, separated into 0.5 mL aliquots and frozen at − 80 ◦C until assayed.

### Determination of human malondialdehyde (MDA) in saliva using an ELISA kit (prepared by Prof. OS)

Saliva samples were centrifuged for 10 min at 4000 xg. The supernatant was separated and used for determination of MDA levels using ELISA Kit Cat No. MBS263626 provided by My BioSource (USA, NY). This kit employs the “Double Antibody Sandwich” technique. The principle of double antibody sandwich is based on the characteristics of a target analytic with more than two possible epitopes that can be identified by both the precoated capture antibody and the detection antibody simultaneously.In this kit, the precoated antibody is an anti-human MDA monoclonal antibody, while the detection antibody is a biotinylated polyclonal antibody. Samples and biotinylated antibodies are added into ELISA plate wells and washed out with PBS or TBS after their respective additions to the wells. Then, avidin-peroxidase conjugates were added to the wells. TMB substrate is used for colouration after the enzyme conjugate has already been thoroughly washed out of the wells by PBS or TBS. TMB reacts to form a blue product from the peroxidase activity, and finally turns yellow after addition of the stop solution (Color Reagent C). The color intensity and quantity of target analytics in the sample are positively correlated [17].

### Sample size calculation

An interventional study by Thomas et al. [55] was used by medical biostatistics unit members, Faculty of Dentistry, Cairo University, to calculate sample size using an independent t-test. The mean and standard deviation for group 1 = 1.36 ± 1.11 while for group 2 = 2.47 ± 0.841,the alpha level of significance = 0.05, and the power of the study was 0.8.The sample size produced was 28 in both groups and increased by 20% to 34 (17 per group) to compensate for drop-outs.

### Randomization and allocation concealment

Simple randomization was generated using www.randomizer.org and performed by the principal investigator. Allocation concealment was performed by placing the treatment assignment in sequentially numbered, opaque, sealed envelopes.

### Masking/blinding

Neither the statistician nor clinical outcome assessor (associate prof. AH) were aware of which medication was being administered, thus yielding a double-blind controlled study.

### Data collection and statistical analysis

All data collected from patients using clinical parameters were recorded electronically for statistical analysis. Categorical data are presented as frequencies (n), and percentages (%), and the chi square test was used for the analysis. Quantitative data were explored for normality using Kolmogorov–Smirnov and Shapiro–Wilk tests and are presented as the mean and standard deviation (SD). Parametric data of age, lesion size and salivary level of MDA were analyzed using independent t test for intergroup comparisons and repeated measures ANOVA followed by Bonferroni post hoc test. VAS data showed a nonparametric distribution so they were analyzed using the Mann -Whitney U test for intergroup comparisons and the Friedman test of repeated measures for intragroup comparisons. When the Friedman test was significant, it was followed by multiple pairwise comparisons utilizing the Wilcoxon signed rank test with Bonferroni correction. The significance level was set at *P* ≤ 0.05 for all tests. Statistical analysis was performed with IBM® SPSS® (SPSS Inc., IBM Corporation, NY, and USA) Statistics Version 26 for Windows.

## Results

**Fig. 4:**
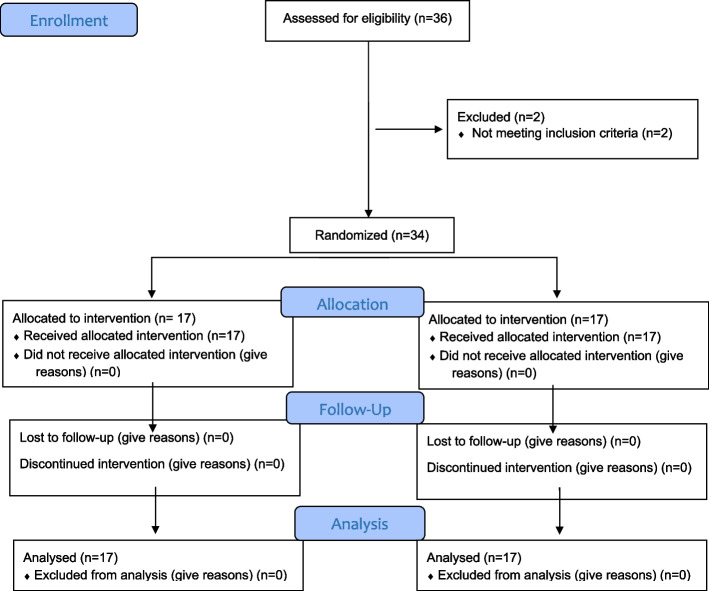
CONSORT flow diagram of participants

During the recruitment phase, 36 patients were assessed for eligibility from September 2019 to February 2022. Two patients did not meet the inclusion criteria due to their chronic systemic diseases. Only thirty-four participants were eligible for inclusion. All patients gave written informed consent and were randomly allocated equally to the intervention group (*n* = 17), who received topical mucoadhesive tablets, and the control group (*n* = 17), who received topical corticosteroids. No participants were excluded during the follow up period (4 weeks) and all participants were analyzed, (Fig. 4). The mean ± SD value of the ages in the intervention group was 35.82 ± 8.36 and for the control group it was 38.41 ± 7.45. There was no significant difference between the ages of the participants in both groups (*P* = 0.348). All the participants in the intervention group were females. In the control group, two (11.8%) of the participants were males, while fifteen (88.2%) were females. There was no significant difference in gender distribution between the groups (*P* = 0.485), (Table 1). Regarding the clinical characteristics of symptomatic OLP lesions, the atrophic form of OLP occurred in 64.7% of the participants in the intervention group and 52.9% of the participants in the control group, while the erosive form of OLP occurred in 35.3% of the participants in the intervention group and 47.1% of the participants in the control group (Table 2).

**Table 1:** Demographic data of age and gender

Groups	Gender				Age (Mean ± SD)
	Male		Female		
	(n)	%	(n)	%	
Intervention	(0)	0.0%	(17)	100.0%	35.82 ± 8.36
Control	(2)	11.8%	(15)	88.2%	38.41 ± 7.45
*P*-value	0.485				0.348S

**Table 2:** Clinical characteristics of symptomatic OLP lesions in both groups

Groups	Atrophic OLP(n)		Erosive OLP(n)	
	(n)	%	(n)	%
**Intervention**	11	64.7%	6	35.3%
**Control**	9	52.9%	8	47.1%

After the 4 week follow up period, both the intervention group and control group showed a significant difference in VAS scores at different follow-up intervals (*p* < 0.001). The highest (mean ± SD) value of VAS was recorded at (week) (7.00 ± 1.06) in the intervention group and (7.41 ± 1.00) in the control group, while the lowest value was found at (4 weeks), which was (2.06 ± 1.92) in the intervention group and (1.94 ± 2.08) in the control group. Pairwise comparisons showed values measured at (week) to be significantly higher than values measured at other intervals except for (2 weeks) (*p* < 0.05) in both groups. Intergroup comparison showed that, at 4 weeks, (the mean ± SD) value of VAS scores in the intervention group was slightly higher, while for other follow-up intervals, the control group was higher; however, the differences did not reach the level of significance (*P* > 0.05) (Table 3).

**Table 3:** Mean and standard deviation (SD) of VAS scores in both groups and different follow-up intervals

Follow-up intervals	(VAS) (Mean ± SD)		*P*-value
	Intervention	Control	
Week	7.00 ± 1.06^A^	7.41 ± 1.00^A^	0.222 ns
2 weeks	5.18 ± 1.13^AB^	5.76 ± 0.90^AB^	0.159 ns
3 weeks	4.00 ± 1.22^BC^	4.06 ± 1.64^BC^	0.858 ns
4 weeks	2.06 ± 1.92^C^	1.94 ± 2.08^C^	0.787 ns
*P*-value	< 0.001*	< 0.001*	

Different superscript letters within the same column indicate a statistically significant difference*; significant (*p* ≤ 0.05) ns; nonsignificant (*p* > 0.05)

**Fig. 5:**
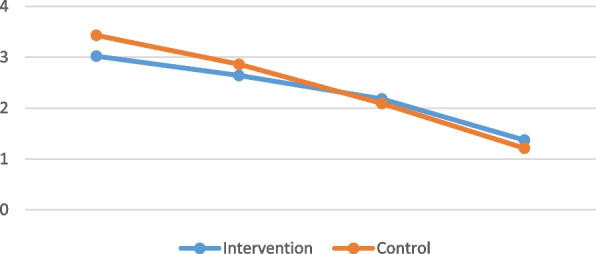
Line chart showing average lesion size in (Thongprasom scale) in different follow-up intervals

Regarding the (mean ± SD) value of lesion size using the (Thongprasom scale), the intervention group showed a significant difference between lesion sizes at different follow-up intervals (*p* < 0.001). The highest (mean ± SD) value of lesion size was recorded at the first week (3.02 ± 0.87), while the lowest value was found at 4 weeks (1.37 ± 0.74). Pairwise comparisons showed that the value measured after one week was significantly higher than the values measured at other intervals except for 2 weeks (*p* < 0.05). In the control group, there was a significant difference between lesion sizes at different follow-up intervals (*p* < 0.001). The highest (mean ± SD) value of lesion size was recorded at week (3.43 ± 0.74), while the lowest value was found at 4 weeks (1.21 ± 0.90). Pairwise comparisons showed that the differences between values of follow-up weeks were all statistically significant (*p* < 0.05). Intergroup comparison showed that, at (3 weeks) and (4 weeks), the (mean ± SD) value of the intervention group was slightly higher than that of the control group, while for other follow-up intervals (week and 2 weeks), the control group was higher. At all follow-up intervals, there was no significant difference between the groups (*P* > 0.05) (Table 4 and Fig. 5).

**Table 4:** Mean and standard deviation of lesion size in (Thongprasom scale) in both groups and different follow-up intervals

Follow-up intervals	Lesion size in thongprasom scale (Mean ± SD)		*P*-value
	Intervention	Control	
Week	3.02 ± 0.87^A^	3.43 ± 0.74^A^	0.148 ns
2 weeks	2.64 ± 0.79^AB^	2.86 ± 0.79^B^	0.410 ns
3 weeks	2.18 ± 0.56^B^	2.09 ± 0.80^C^	0.692 ns
4 weeks	1.37 ± 0.74^C^	1.21 ± 0.90^D^	0.564 ns
*P*-value	< 0.001*	< 0.001*	

Different superscript letters within the same column indicate a statistically significant difference*; significant (*p* ≤ 0.05) *ns* nonsignificant (*p* > 0.05)

**Fig. 6:**
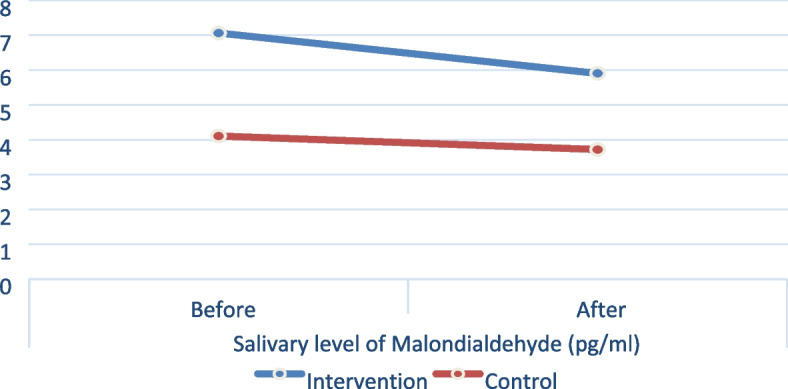
Line chart showing the average salivary level of malondialdehyde (pg/ml) at different follow-up intervals

In addition to the clinical assessment, the salivary level of malondialdehyde (pg/ml) before and after the treatment in both groups was assessed which showed that the intervention group value of salivary level of malondialdehyde (pg/ml) measured before (7.08 ± 4.46) was higher than value measured after treatment (5.92 ± 2.73), however the difference was not statistically significant (*p* = 0.311). Additionally the control group showed that the salivary level of malondialdehyde (pg/ml) measured before treatment (4.12 ± 2.37) was higher than the value measured after treatment (3.73 ± 2.25) which was not statistically significant (*p* = 0.522). At both intervals, the (mean ± SD) value of the intervention group was significantly higher than that of the control group (*P* < 0.05) (Table 5 and Fig. 6).

**Table 5:** Mean and standard deviation of salivary level of malondialdehyde (pg/ml) in both groups and different follow-up intervals

Follow-up intervals	Salivary level of Malondialdehyde (pg/ml) (Mean ± SD)		*P*-value
	Intervention	Control	
Before	7.08 ± 4.46	4.12 ± 2.37	0.022*
After	5.92 ± 2.73	3.73 ± 2.25	0.016*
*P*-value	0.311 ns	0.522 ns	

*ns* nonsignificant (*p* > 0.05)

Finally, PGI-C assessing patient experience with their OLP at end of dosing also showed clinically meaningful improvements in both groups with 12/17 patients (70.5%) in mucoadhesive Coq10 intervention group and 14/17 patients (82.3%) in the control group reporting their OLP feeling much better or very much better.

Regarding drug safety in both groups, none of the participants in either group reported any temporary or permanent adverse effects with either treatment during the 4 week follow up period.

## Discussion

The chronic nature of OLP, prolonged course of treatment, and frequent exacerbation of the condition increase the incidence of steroid side effects [15], therefore, the search for new treatment modalities has become essential to overcome the side effects of the long term use of steroids. Among these, antioxidant and anti-inflammatory agents were proposed based on the role that might be played by oxidative stress in the pathogenesis of OLP [5] and [33].

In addition to the antioxidant and anti-inflammatory effects of CoQ10, its topical use in the form of mucoadhesive tablets in the present study has many advantages including intimate contact with the target mucous membrane, sustained drug release, increased drug absorption, and bioavailability, avoidance of enzymatic degradation in the GIT, and decreased adverse drug effects [49]. Additionally, the systemic use of CoQ10 was reported in a few cases to cause mild insomnia, rashes, nausea, and upper abdominal pain [39]. In our study, the topical use of CoQ10 prevented the incidence of these side effects.

The mucoadhesiveness of these tablets is gained from the use of mucoadhesive carbapol polymer that rapidly swells when touching the target area, thus providing sustained and controlled release of the drug from 6–8 h, and a strong mucosal adhesion effect [42]. Furthermore, its topical use is safe with a nonsensitizing effect and no effect on the biological activity of other drugs [40]. Anhydrous lactose was added to these tables to improve taste, with no effect on the biological activity of the drug used [21].

Up to our knowledge, the current study is the first randomized control clinical trial evaluating the effectiveness of topical use of CoQ10 in the management of symptomatic OLP. Consequently, no similar previous studies are available for comparison with our results. Shoukheba and Elgendy’s [54] study is the only one where CoQ10 was tried for the management of OLP, but in the systemic form of 30 mg CoQ10 capsules, combined with topical corticosteroid.

The results of the current study showed that the topical use of CoQ10 mucoadhesive tablets significantly reduced both pain sensation and clinical signs with maximum clinical improvement at the fourth week and no significant differences when compared with the results of topical corticosteroid. The effective role of the topical use of CoQ10 was also seen in the study conducted by Shoukheba and Elgendy [54], who reported that the systemic use of CoQ10 in combination with topical corticosteroids improved the condition more than the topical use of corticosteroids alone.

In addition to the clinical assessment, the salivary levels of MDA in both groups decreased after treatment with no significant difference. Therefore, we could assume that the clinical improvement in both groups might be due to the anti-inflammatory effect of both corticosteroids [12] and CoQ10 [8, 19], which directly have a great effect on decreasing the secretion of proinflammatory cytokines such as TNF-α and subsequently, oxidative stress damage [18, 35].

However, it is expected that corticosteroids would have a more potent anti-inflammatory effect than CoQ10. Thus, it seems that while the decrease in oxidative stress in the case of triamcinolone could be totally a result, in the case of CoQ10, it is partly a result and partly due to the powerful antioxidant role of CoQ10 which was previously detected by Ushikoshi-Nakayama et al. [58]. This double action of CoQ10 could be the reason for its effect being equivalent to that of triamcinolone.

Topical CoQ10 has been previously investigated in other oral conditions, such as periodontal and gingival conditions, and showed a great clinical reduction in the inflammatory condition after a few weeks [13, 44, 45, 48].

Comparing our study with other studies using antioxidant agents in the management of OLP, such as selenium -ACE [6], selenium [46], Aloe vera (AV) [1], curcumin [47], lycopene [50], quercetin [3] and ozone therapy [34],we can deduce that, coQ10 can be used as an alternative treatment or in combination with corticosteroids for OLP management, similar to other antioxidants, excluding quercetin which did not show any significant difference when added to topical corticosteroids compared with the placebo treatment [3]. In addition, coQ10 was sufficient to improve the oral condition clinically when taken in daily small doses for a short period (4 weeks), unlike curcumin, which improved the oral condition after being taken in large amounts for a long period [47]. Only a few studies have measured salivary MDA levels, including selenium [46], and curcumin [47], which showed the antioxidant effect of systemic use of selenium and curcumin, as seen with the topical use of coQ10 in our study. The study was limited by the short follow-up, precluding the opportunity to evaluate the relapse rate and the effect of topical use of CoQ10 when used for a long duration. Furthermore, the small sample size recommended the need for more clinical trials to conclude the effective role of CoQ10 in the management of symptomatic OLP.

## Conclusions

1. Topical application of mucoadhesive CoQ10 tablets on symptomatic OLP lesions leads to significant pain relief and clinical improvement of the condition in addition to decreasing the salivary levels of one of the markers of oxidative stress (MDA).
2. CoQ10 in a mucoadhesive formula is as effective as the standard treatment triamcinolone acetonide in reducing pain scores and lesion size in OLP**.**
3. Topical CoQ10 as an antioxidant and anti-inflammatory agent together with its analgesic effect is a safe treatment modality for symptomatic OLP, with no apparent side effects**.**

## Recommendations

1. Studies with larger sample sizes are needed to conclude the effective role of CoQ10 in the management of symptomatic OLP.
2. Different concentrations of CoQ10 need to be used to reach the optimum dose required to achieve optimum management of OLP with no side effects.
3. The period between lesion remission and exacerbation for CoQ10 should be measured.
4. Evaluation of CoQ10 use for a long duration is also needed.

## Data Availability

The datasets used and/or analyzed during the current study are not publicly available due [for better patient data confidentiality] but are available from the corresponding author on reasonable request.

## Abbreviations

OLP: Oral lichen planus
CoQ10: Co enzyme Q10
ROS: Reactive oxygen species
TNF-α: Tumor necrosis factor-alpha
NF-Kb: Nuclear factor kappa-light-chain-enhancer of activated B cells
OS: Oxidative stress
ICAM: Intercellular adhesion molecule
VCAMs: Vascular adhesion molecules
MDA: Malondialdehyde
